# Formulation Development and Characterization of Quercetin Solid Dispersions with Integrated In Vitro Release and In Vivo Pharmacokinetic Assessment

**DOI:** 10.3390/ph19101625

**Published:** 2026-10-13

**Authors:** Zi-Xuan Guo, Zi-Yang Li, Xia-Li Wei, Xin-Yu Ji, Ting-Ting Lian, Fei Zhu, Qing-Ri Jin

**Affiliations:** 1 School of Pharmacy, Zhejiang Provincial People’s Hospital (Affiliated People’s Hospital), Hangzhou Medical College, Hangzhou 311399, China; 2 Key Laboratory of Applied Technology on Green-Healthy Animal Husbandry of Zhejiang Province, Zhejiang Provincial Engineering Laboratory for Animal Health Inspection & Internet Technology, College of Animal Science and Technology & College of Veterinary Medicine, Zhejiang A & F University, Hangzhou 311300, China; 3 College of Veterinary Medicine, Zhejiang A & F University, Hangzhou 311300, China

**Keywords:** quercetin, solid dispersion, solubility, bioavailability, pharmacokinetics

## Abstract

**Purpose:** To address the challenges of low water solubility and poor bioavailability of quercetin (QUE), which limit its clinical application, and to develop an optimized oral solid dispersion formulation to enhance its absorption. **Methods:** Quercetin solid dispersions (QUE-SD) were prepared using the solvent evaporation method. The study involved screening single-factor and combined carriers, followed by formulation optimization via orthogonal analysis based on solubility and cumulative dissolution rates. The optimized dispersions were characterized using X-ray diffraction (XRD), scanning electron microscopy (SEM), differential scanning calorimetry (DSC), and Fourier transform infrared spectroscopy (FTIR). Additionally, transmembrane transport was evaluated using Caco-2 and MDCK cell models, and oral bioavailability was assessed through pharmacokinetic studies in rats. **Results:** The optimal formulation was determined to be a 1:3:1 ratio of Quercetin, Hypromellose E5, and polyvinylpyrrolidone K13-18, with an evaporation temperature of 55 °C and a mixing time of 4 h. In cell transport models, the apparent permeability coefficient of QUE-SD increased by 1.14 (Caco-2) and 1.1 (MDCK) times. Pharmacokinetic data confirmed that the solid dispersion significantly improved both the absorption rate and oral bioavailability of quercetin in rats. **Conclusions:** The prepared quercetin solid dispersion effectively enhances the water solubility, transmembrane transport, and systemic absorption of quercetin. These findings provide a viable strategy and theoretical basis for developing high-bioavailability oral formulations of quercetin.

## 1. Introduction

Quercetin (QUE) is a natural flavonoid compound [1,2,3,4], It has very low aqueous solubility but is soluble in ethanol and lipids [5,6,7]. Quercetin glycosides are formed by attaching sugar groups (such as glucose and rhamnose) to -OH groups. The attached sugar groups can alter their solubility, absorption, and in vivo effects [8,9,10]. In the small intestine, certain QUE glycosides can be hydrolyzed by intestinal β-glucosidases to release the QUE aglycone [11,12,13,14,15,16,17]. Quercetin absorbed across the small-intestinal epithelium undergoes extensive phase II conjugation, including glucuronidation, sulfation, and methylation, whereas unabsorbed quercetin and its glycosides can reach the distal intestine and colon, where they are degraded by the gut microbiota into lower-molecular-weight phenolic acids [12,18,19]. QUE has a half-life of 11–28 h, thus requiring continuous administration to maintain its effective blood concentration [15,20,21].

QUE binds to salivary proteases after oral administration to form QUE-protein conjugates [22,23], but does not affect absorbing QUE [24]. Gastric acid is present in the stomach, where it can convert QUE into protocatechuic acid [25,26,27]. Upon entering the small intestine, QUE undergoes two major metabolic pathways: first, QUE undergoes glucuronidation by uridine diphosphate glucuronosyltransferase (UDPGT) to produce glucuronide derivatives. Second, catechol-O-methyltransferase (COMT) converts QUE to O-methylated products [28,29]. The respiratory and excretory systems eliminate the breakdown products of QUE, which include carbon dioxide and phenolic acids, Rangel-Ordóñez, L et al. have found that when QUE is administered orally, the body eliminates the majority of it as carbon dioxide (52.1%), through urine (4.6%), and in feces (1.9%) [30]. However, due to the rapid metabolism and poor water solubility, its bioavailability after oral administration is extremely low [31]. Therefore, improving the bioavailability of QUE as a prospective therapeutic agent remains a challenge for research and drug development [32].

Because the poor aqueous dissolution and extensive first-pass metabolism of QUE limit its oral bioavailability, appropriate formulation strategies are needed to improve its oral delivery. Oral formulations of QUE facilitate long-term intake with high compliance. Earlier studies showed that healthy adults consuming 1095 mg of QUE with a standardized meal could reach a plasma concentration of 1430 nmol/L within 24 h [33]. Another benefit of oral formulations of QUE is the ease with which repeated dosing can be achieved. PK study in rats showed that after oral administration of 600 mg/kg of EGb 761^®^, plasma concentrations of QUE reached approximately 582 nmol/L. However, the plasma concentration of QUE increased 4.6-fold after repeated administration of the drug [34]. Prolonged ingestion of QUE does not accumulate in any tissue compared to a single administration [35]. The metabolism of QUE in the small intestine and stomach results in less than 10% bioavailability in the body [36].

Amorphous solid dispersion (ASD) is a technique for dispersing a drug (active pharmaceutical ingredient, API) in a carrier [37]. Drugs in ASDs accelerate dissolution mainly by presenting them in an amorphous state that does not need to overcome the lattice energy required for the dissolution of crystalline drugs [38]. The amorphous state has higher free energy and apparent water solubility compared with the crystalline structure. ASD improves oral bioavailability and reduces pharmacokinetic variability [39]. M. Kakran et al. [40] used nanosuspension evaporative precipitation (EPN) to formulate QUE nanoparticles, inclusion complexes, and solid dispersions of QUE using polyvinylpyrrolidone and Poloxamer F127 is the carrier that improve the solubility of QUE reference. The solubility of QUE solid dispersions was higher than inclusion compounds at lower carrier concentrations. Bing Chen [41] et al. prepared amorphous solid dispersions of bioflavonoids with polyvinylpyrrolidone K30, which showed an increase in the solubility of the solid dispersions compared with that of the API. YiRu Wang [42] et al. fabricated amorphous solid QUE by co-precipitation method. The experimental results showed that the dissolution behavior of QUE ASD depends on the interaction between the polymer, QUE and the inhibition ability of the polymer crystallization. However, the pharmacokinetic effect of the polymer in rats has not been clarified.

In this study, QUE solid dispersions will be prepared by the solvent evaporation method to improve the solubility, dissolution rate and in vitro uptake efficiency and will be subjected to the optimization of the preparation process. The successful conduct of this experiment will provide data support and a theoretical basis for the clinical application of QUE solid dispersions.

## 2. Results

### 2.1. Solubility Measurement Results

**Figure 1:**
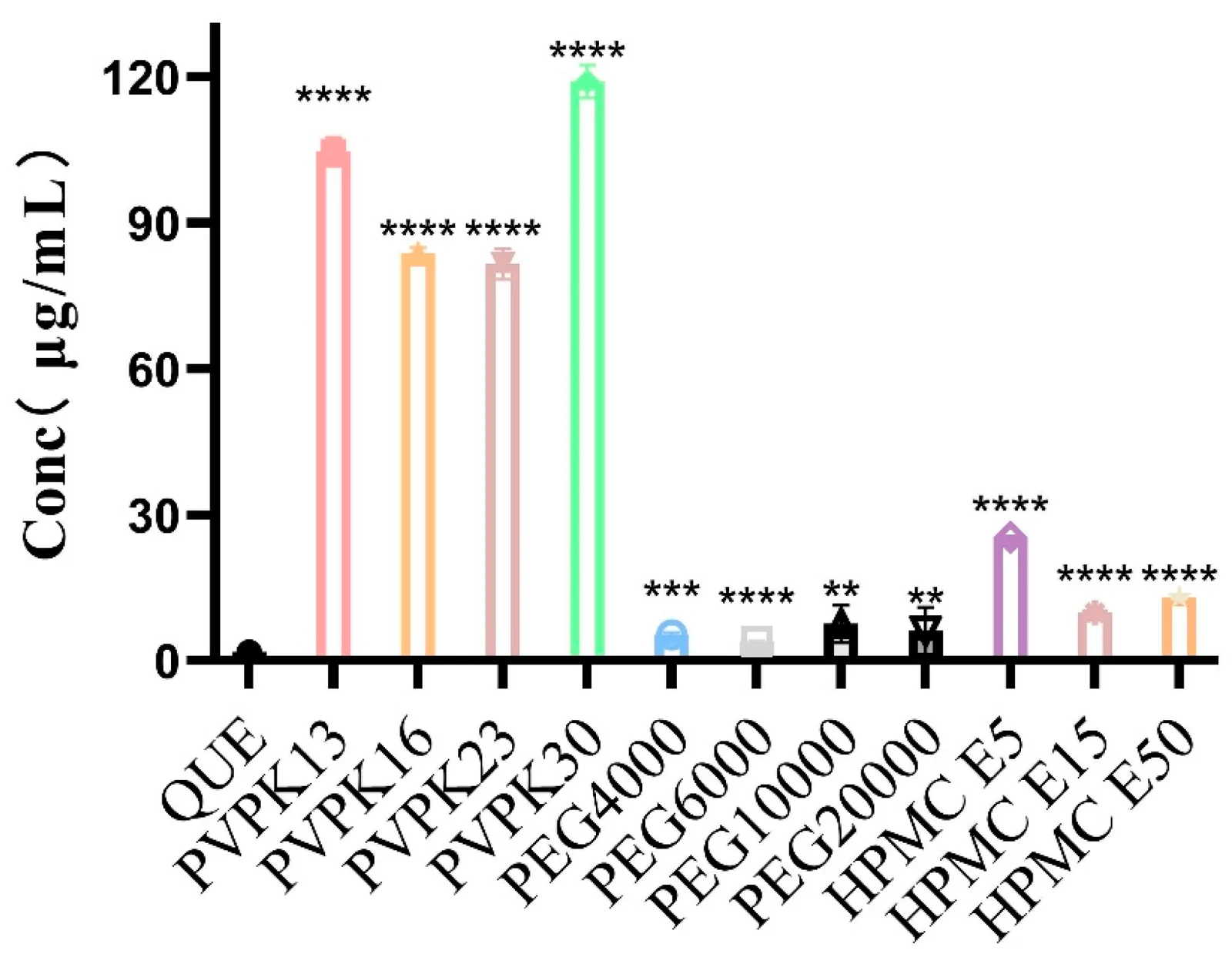
Solubility for pure quercetin (QUE) and single-carrier samples (*n* = 3). ** *p* < 0.01, *** *p* < 0.001, and **** *p* < 0.0001 compared with pure QUE.

**Figure 2:**
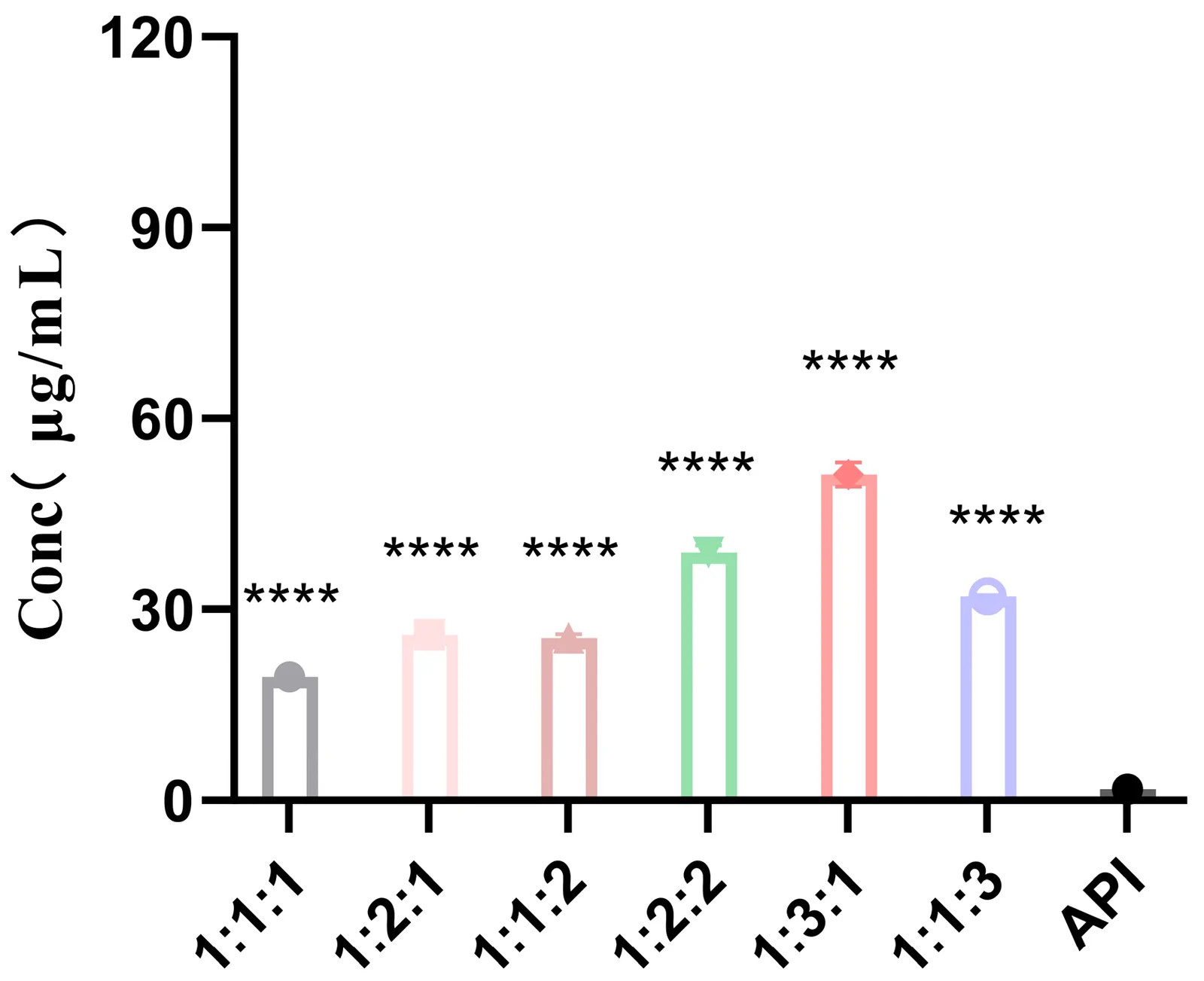
Solubility for quercetin (QUE) and combined-carrier samples (*n* = 3). **** *p* < 0.0001 compared with the API group.

As shown by the experimental results in Figure 1 and Figure 2, the aqueous solubility of QUE at 37 °C was only 1.78 ± 0.17 µg/mL. Owing to the presence of the carrier materials, the solubilities of the physical mixture (quercetin physical mixture, QUE-PM) and QUE-SD were significantly higher than that of unprocessed QUE. Among the carriers investigated, the formulations containing PVP-K13 and PVP-K30 showed significantly higher solubility than those containing the other carriers. However, PVP-K30 was more viscous than PVP-K13, and the solid dispersion remained tacky after drying for 24 h, making the grinding process complicated and time-consuming. Therefore, PVP-K13 and HPMC were selected as the combined carriers for the subsequent experiments. As shown in Figure 2, the formulation with a QUE E5 mass ratio of 1:3:1 exhibited the highest solubility (51.27 µg/mL), representing a 28.8-fold increase compared with unprocessed QUE, and its dissolution rate was higher than that of the 1:1:3 formulation.

### 2.2. Cumulative Dissolution Rate Test

#### 2.2.1. Cumulative Dissolution Profiles of Single-Carrier and Combined-Carrier Systems

**Figure 3:**
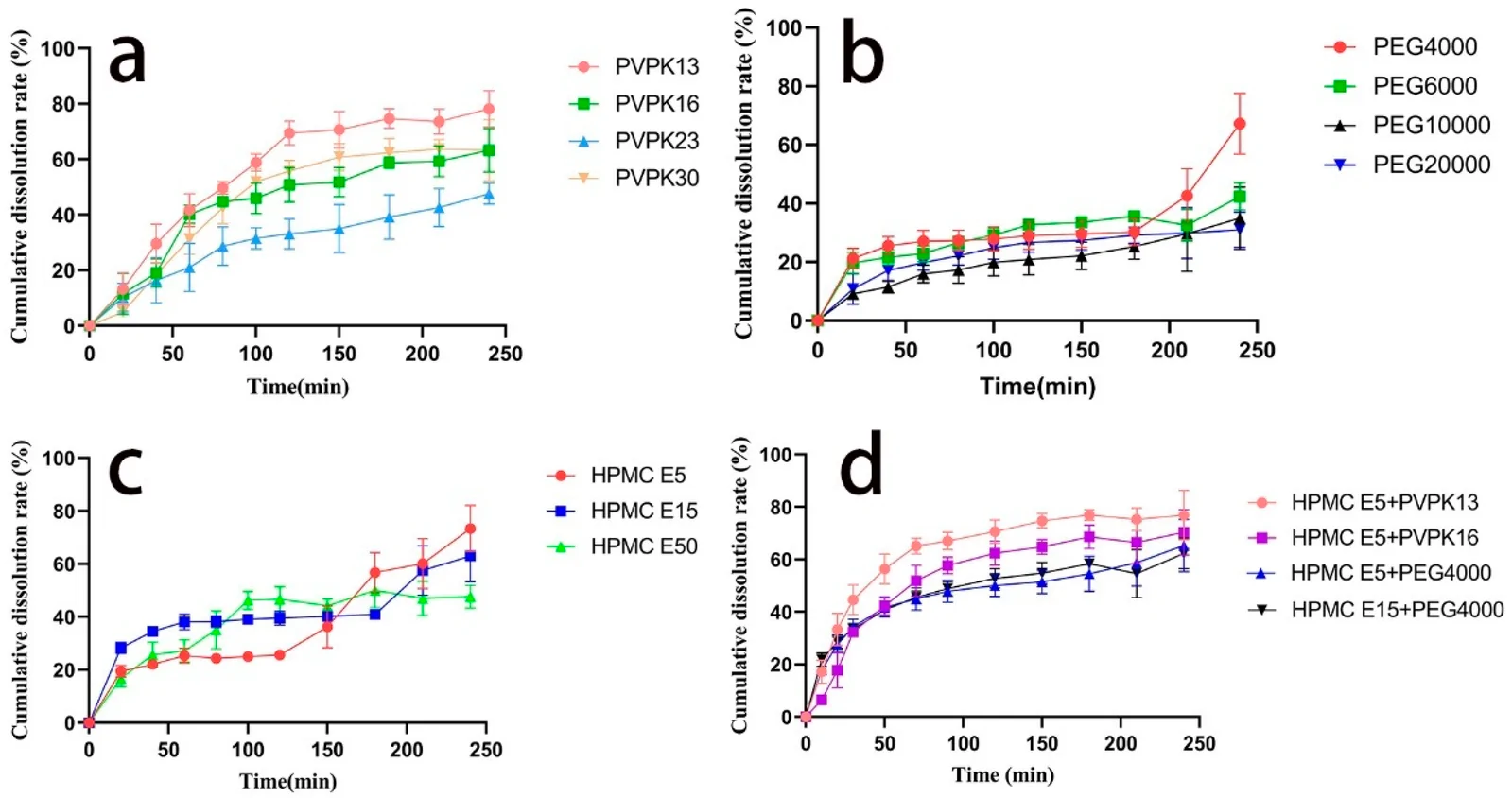
Dissolution curves of solid dispersion samples in distilled water (*n* = 6). (**a**) PVPK series of quercetin solid dispersions; (**b**) PEG series of solid dispersions prepared by solvent evaporation method; (**c**) HPMC series of quercetin solid dispersions. (**d**) HPMC screening vectors.

As shown in Figure 3a, the solid dispersion prepared using PVP-K13 as the carrier exhibited the highest cumulative dissolution. As shown in Figure 3b, the cumulative dissolution of the solid dispersion prepared using PEG 4000 as the carrier by the solvent evaporation method reached 70% at 4 h. As shown in Figure 3c, the solid dispersion prepared using HPMC E5 as the carrier showed higher cumulative dissolution at 4 h than those prepared using the other two HPMC carriers. Based on the results shown in Figure 3a–c, PVP-K13 and HPMC E5, which provided the most favorable dissolution performance, were selected as the combined-carrier system for the preparation of QUE-SD. As shown in Figure 3d, the combination of HPMC E5 and PVP-K13 showed the strongest synergistic effect, with cumulative dissolution reaching 40% at 40 min, indicating that the combined-carrier system accelerated the dissolution of QUE in water.

#### 2.2.2. Cumulative Dissolution Profiles of Combined-Carrier Systems with Different Mass Ratios

**Figure 4:**
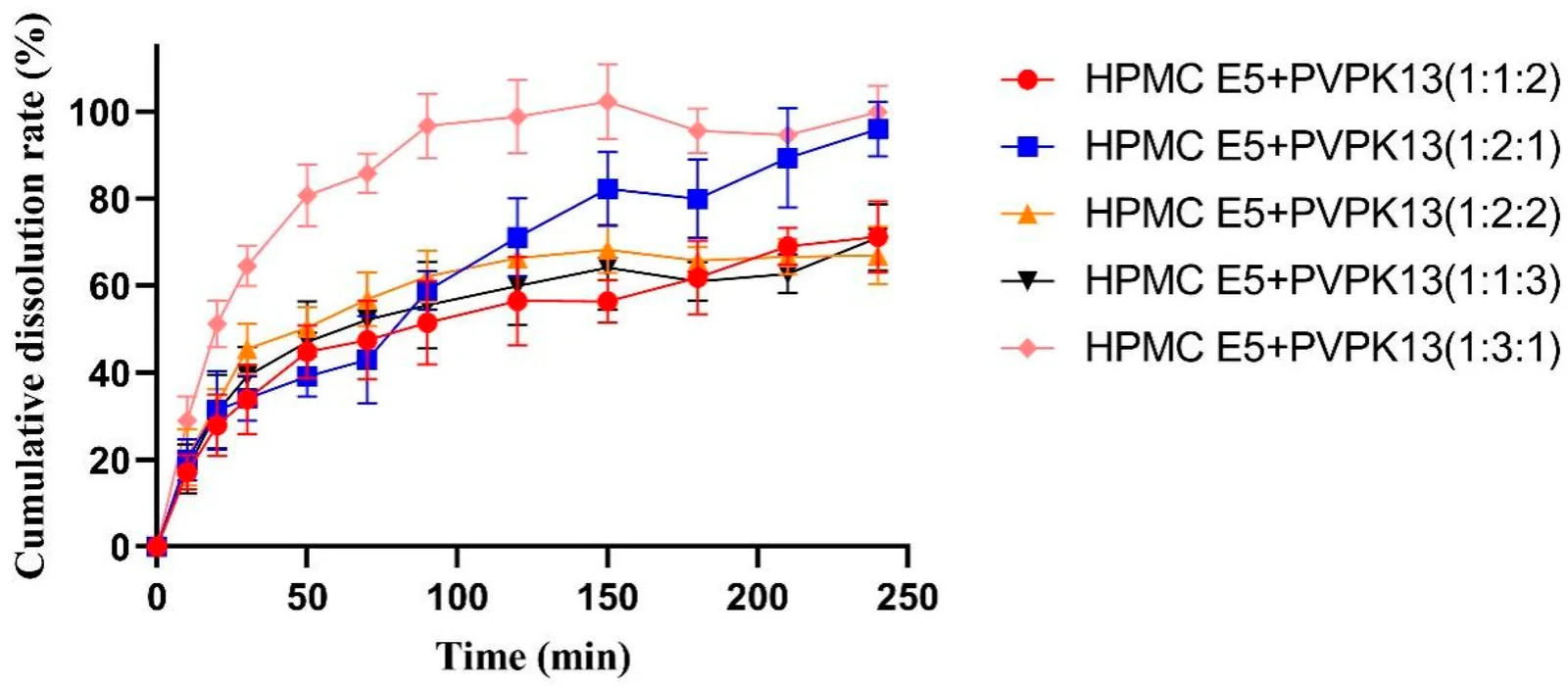
Dissolution curves of solid dispersion samples (*n* = 6).

Because the solid dispersions prepared using single carriers showed slower dissolution than those prepared using combined carriers, HPMC was added to PVP-K13 to prepare a combined-carrier solid dispersion. HPMC is widely used in pharmaceutical formulations because of its solubility in cold water, biocompatibility, and viscosity. As shown in Figure 4, the formulation with a QUE E5 mass ratio of 1:3:1 exhibited the most favorable dissolution performance, with the cumulative dissolution of QUE reaching 60% at 30 min, further supporting the results of the single-carrier screening. Therefore, the QUE E5 mass ratio of 1:3:1 was selected for subsequent optimization of the preparation process.

#### 2.2.3. Orthogonal Experimental Design for Preparation Technology

As shown in Table 1, a two-factor, three-level orthogonal design was used to investigate the effects of stirring time (factor A) and rotary evaporation temperature (factor B) on the cumulative dissolution of QUE-SD. The best result of the experiment was A1B3, and the stirring time had a greater effect on the cumulative dissolution rate of the solid dispersion than the rotary evaporation temperature. At stirring times of 4 and 12 h, the cumulative dissolution obtained at 55 °C was higher than that obtained at 35 and 45 °C. However, extending the stirring time to 24 h did not further improve the cumulative dissolution, indicating that prolonged stirring was not beneficial under the investigated conditions. Therefore, the best preparation process was set as follows, the temperature was 55 °C, the stirring time was 4 h, and the cumulative dissolution rate was 101.0%.

### 2.3. Characterization of Solid Dispersions

#### 2.3.1. SEM and XRD

**Figure 5:**
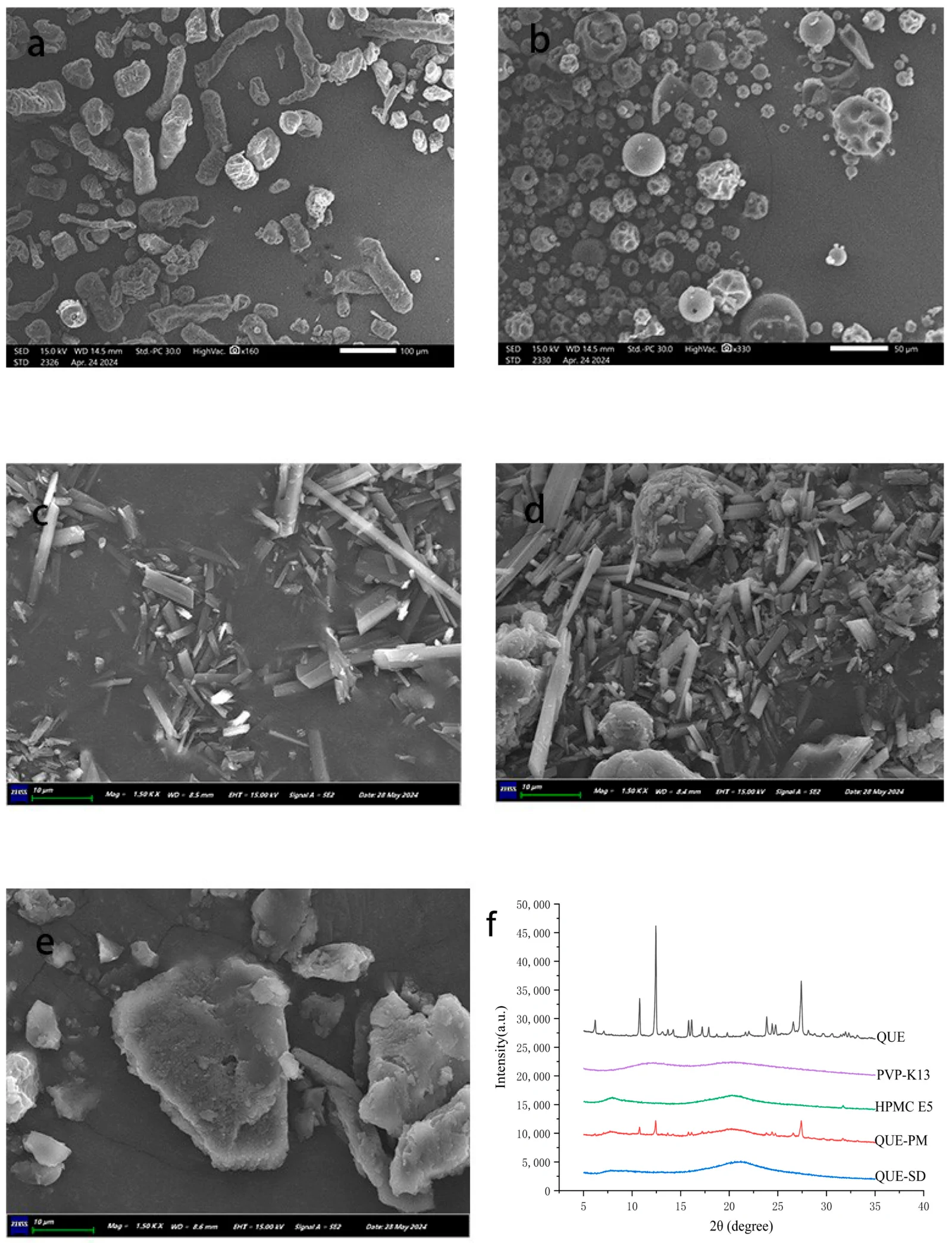
SEM patterns of HPMC E5 (**a**), PVP-K13 (**b**), QUE (**c**), QUE-PM (**d**), and QUE-SD (**e**). X-ray diffraction patterns of QUE, PVP-K13, HPMC E5, QUE-PM, and QUE-SD (**f**).

To investigate the morphological differences between the samples, they were analyzed by SEM, and the results are shown in Figure 5. The SEM image of QUE showed clusters of irregularly arranged rod-like crystals, while HPMC E5 showed a cylindrical structure with a more regular shape than QUE. PVP-K13 showed a homogeneous spherical shape. The SEM image of QUE-PM showed visible rod-like QUE crystals, indicating that QUE retained its original particle morphology after physical mixing. In contrast, the QUE-SD showed a dense lamellar structure, with neither obvious QUE rod-like structures nor conspicuous carrier cylindrical and spherical structures.

As shown in Figure 5, the X-ray diffractograms of five samples (QUE HPMC E5, PVP-K13, QUE-PM, and QUE-SD) were stacked for visual comparison, and the formation of the solid dispersion was confirmed by detecting the crystal morphology. QUE has high-intensity crystal diffraction peaks at 2θ values at 10.8, 12.36, 26.5, and 27.5°, suggesting a crystal structure is formed [43,44]. Meanwhile, PVP K13 and HPMC E5 do not have any crystal diffraction peaks in this range, showing broad peaks of amorphous material caused by random X-ray scattering. QUE-PM exhibited the same characteristic diffraction peaks as QUE, albeit with lower intensities, suggesting that QUE retained its crystalline state in the physical mixture. The curves of the solid dispersion are comparable to those of the carrier and do not show the crystalline characteristic peaks of QUE. This indicates that, in the solid dispersion system, QUE is molecularly dispersed within the carrier, resulting in the loss of the characteristic peaks, suggesting that QUE exists in an amorphous state in the solid dispersion.

#### 2.3.2. DSC and Thermogravimetric Analysis (TGA)

**Figure 6:**
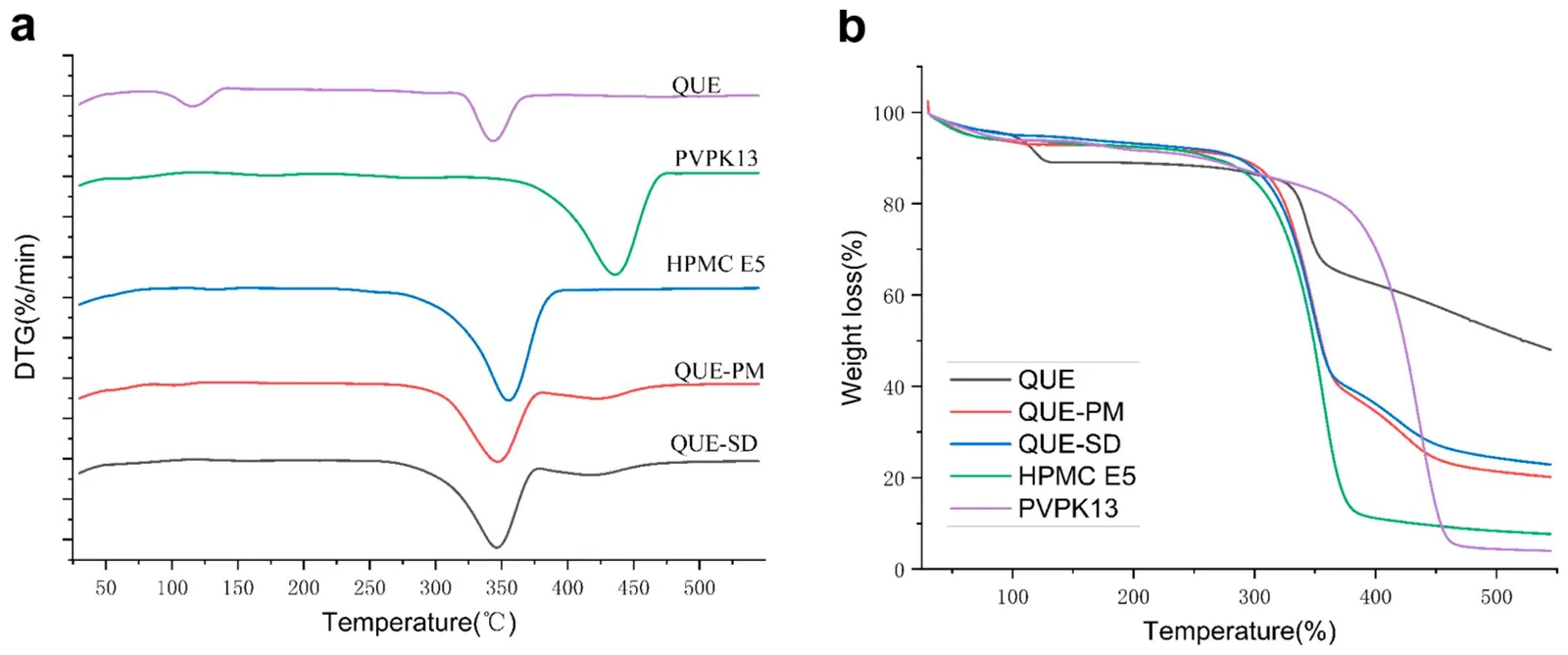
Differential scanning calorimetry curves and thermogravimetric curves of QUE, PVP-K13, HPMC E5, QUE-PM, and QUE-SD: (**a**) differential scanning calorimetry (DSC) curves; (**b**) thermogravimetric analysis (TGA) curves.

The DSC thermograms are shown in Figure 6. QUE contains two kinds of water of crystallization, and there is a water-loss peak at about 116 °C [42]. At 343 °C (the melting point of QUE), there is an obvious exothermic behavior. PVP K13, the polymer compound, had no obvious melting peak and a sharp exothermic peak at 435 °C, which might be formed by the breakage of the polymer chain of PVP K13 at high temperature. Meanwhile, HPMC E5 is wider at 353 °C and the carbonization temperature of HPMC is 280–300 °C, which may be due to the deformation of HPMC E5 at high temperatures and an exothermic peak formed by the breakage of the C ring chain. In the curves of QUE-PM and QUE-SD, the exothermic peak of QUE at 343 °C tends to deviate to the right, which may be due to the mixing of QUE with the HPMC carrier. QUE is loaded in HPMC, resulting in the superposition of the exothermic peaks of HPMC and QUE exhibited, and thus the exothermic peak becomes wider than HPMC and QUE. At 400–500 °C, there is a downward appearance of a large wide peak exhibiting an exothermic feature of PVP-K13, but the exothermic peak was reduced compared with PVP-K13. Combining this with XRD plots showed that QUE solid dispersion was successfully prepared.

A thermogravimetric analyzer was used to study the thermal properties of the samples. As shown in Figure 6, the samples all showed varying degrees of water evaporation when heated to about 275 °C. The residual weights of QUE, PVP-K13, HPMC E5, physical mixture and solid dispersion were 70.30, 82.87, 48.30, 55.69 and 52.64% when the temperature reached 350 °C, respectively. The weight loss of QUE was divided into two stages. The weight loss of QUE occurred in two stages: an initial weight loss of approximately 10% below 125 °C, which was mainly attributable to the loss of crystal water from hydrated QUE, followed by a major weight loss at up to 350 °C due to the thermal decomposition of QUE. Whereas at 450 °C, the weight loss of HPMC E5 and PVP-K13 was about 87 and 94%. It indicated that the two carriers suffered from polymer chain breakage and severe water loss by hydroxyl condensation at 450 °C. The physical mixtures and solid dispersions in QUE-PM and QUE-SD showed better thermal stability due to the presence of QUE. QUE-SD showed better thermal stability than QUE-PM.

#### 2.3.3. Fourier Transform Infrared Spectroscopy (FT-IR)

**Figure 7:**
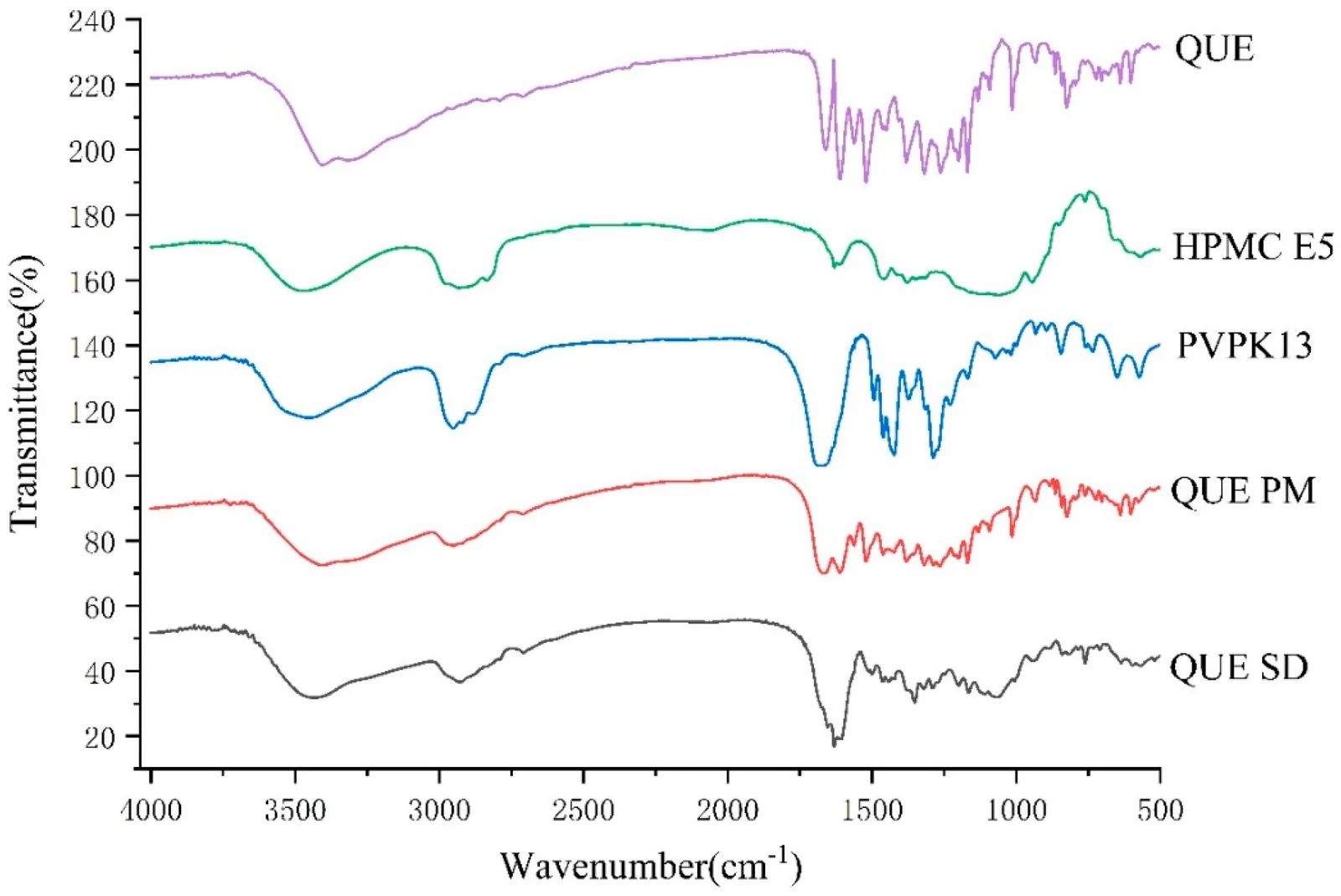
Fourier transform infrared spectroscopy of QUE, PVPK13, HPMC E5, QUE-PM, and QUE-SD.

The FTIR spectra of QUE, HPMC E5, PVPK13, QUE-PM, and QUE-SD are shown in Figure 7. The characteristic peak of QUE at 3379 cm^−1^ is characteristic of -OH in its structure. The absorption peaks at 1667 cm^−1^ are C=O stretching vibration peaks and are C-C stretching absorption peaks at 1610 cm^−1^; the peaks at 1519 cm^−1^ and 1581 cm^−1^ are attributed to C-H bending peaks and benzene ring QUEkbone vibration peaks [45]; the absorption peaks at 1256, 1194, and 1169 cm^−1^ are C-O aryl ether, C-O phenol stretching, and C-CO-C carbonyl stretching and bending peaks; and the absorption peak at 1014 cm^−1^ indicated that the substitution mode in the benzene ring was para-substitution. These results are similar to previous reports [46]. The FTIR spectrum of PVPK13 showed a characteristic -C=O stretching vibration peak at 1684 cm^−1^ [47]. The broader absorption peak at 2900 cm^−1^ of HPMC E5 was caused by -CH_3_ and -CH_2_ stretching vibrations. The physical mixture showed a simple superposition of the characteristic peaks of QUE at 3379 cm^−1^ and carrier at 2945 cm^−1^. Characteristic peaks of QUE appeared as a simple superposition compared to QUE, indicating that QUE-PM did not change the crystalline state of QUE [42]. The peak shape of the absorption peak of QUE-SD became broad and flat from sharp, which was due to the destruction of the crystalline form of QUE. The internal rearrangement of the molecules showed the structure of long-range disordered and short-range ordered, the characteristic peak of QUE disappeared at 1610 cm^−1^–1169 cm^−1^, and the intensity of the characteristic peak of the carrier was weakened, which indicated that the preparation of the solid dispersion system of QUE was successful, which was in agreement with the results of the XRD spectroscopy and the SEM. 

#### 2.3.4. In Vitro Dissolution Study

**Figure 8:**
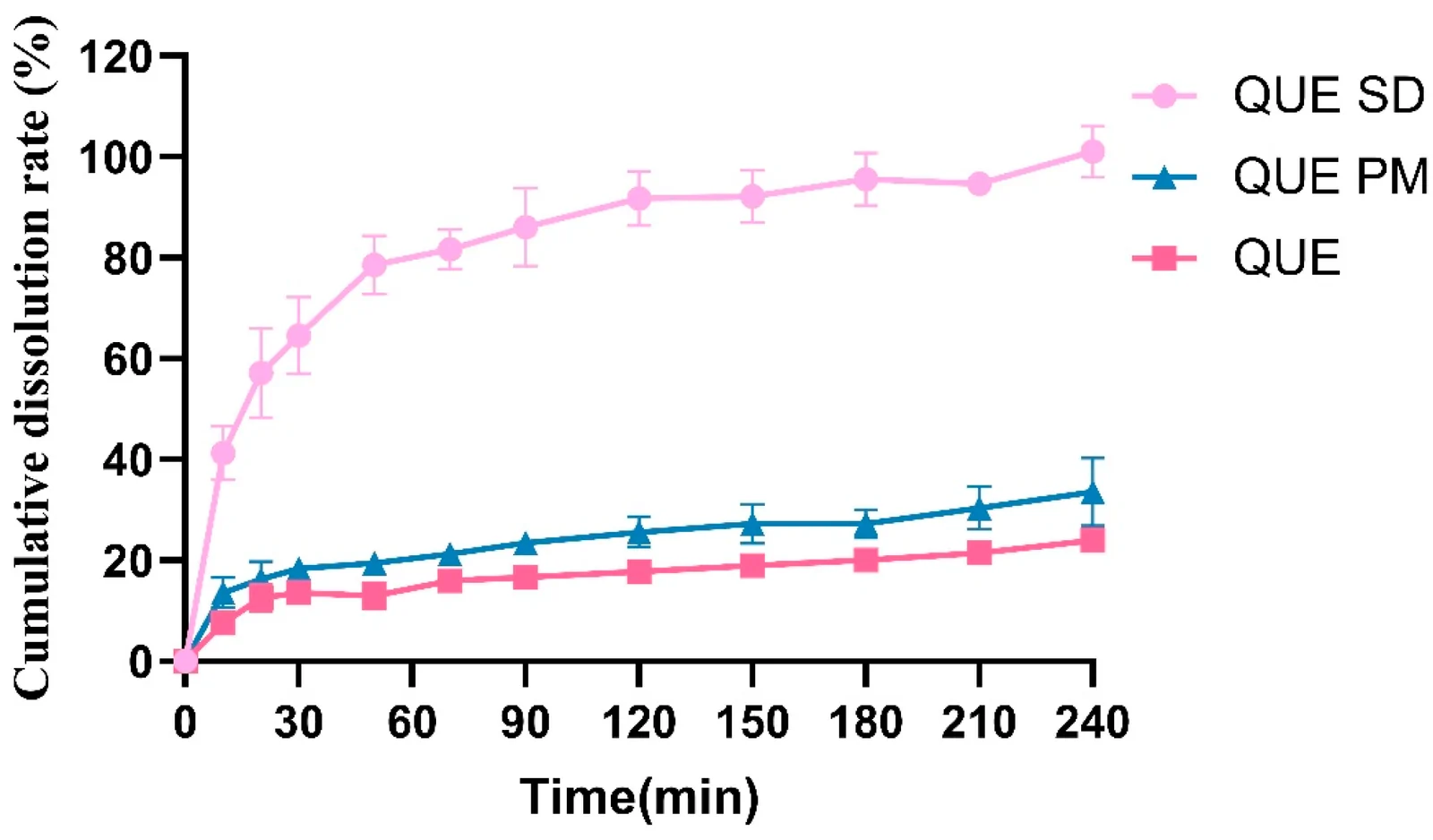
Cumulative dissolution rates of QUE, QUE-PM and QUE-SD.

In the present study, HPMC E5 and PVP K13 were used as polymeric carriers to prepare QUE-SD by the solvent evaporation method. HPMC is a cellulose derivative that is widely used in pharmaceutical formulations because of its water solubility, biocompatibility, transparency, and rheological properties. The dissolution profiles of QUE, QUE-PM, and QUE-SD are shown in Figure 8. Owing to its hydrophobic polyphenolic structure, QUE exhibited limited dissolution, with the cumulative percentage dissolved reaching only approximately 20% at 240 min. After the addition of the carriers, the cumulative percentage dissolved from QUE-PM increased to approximately 30% at 240 min. Nevertheless, because physical mixing did not alter the crystalline state of QUE, the improvement was limited to approximately 10 percentage points. QUE-SD prepared by the solvent evaporation method exhibited an altered solid state, with QUE predominantly dispersed in an amorphous form within the carrier matrix. This change reduced the crystal-lattice energy barrier associated with dissolution and increased the cumulative percentage dissolved. The cumulative percentage dissolved from QUE-SD reached approximately 100% at 150 min. These results indicate that the solid dispersion significantly improved the dissolution performance of QUE.

### 2.4. Cell Test

#### Cell Cytotoxicity Test and Transmembrane Transport Test

**Figure 9:**
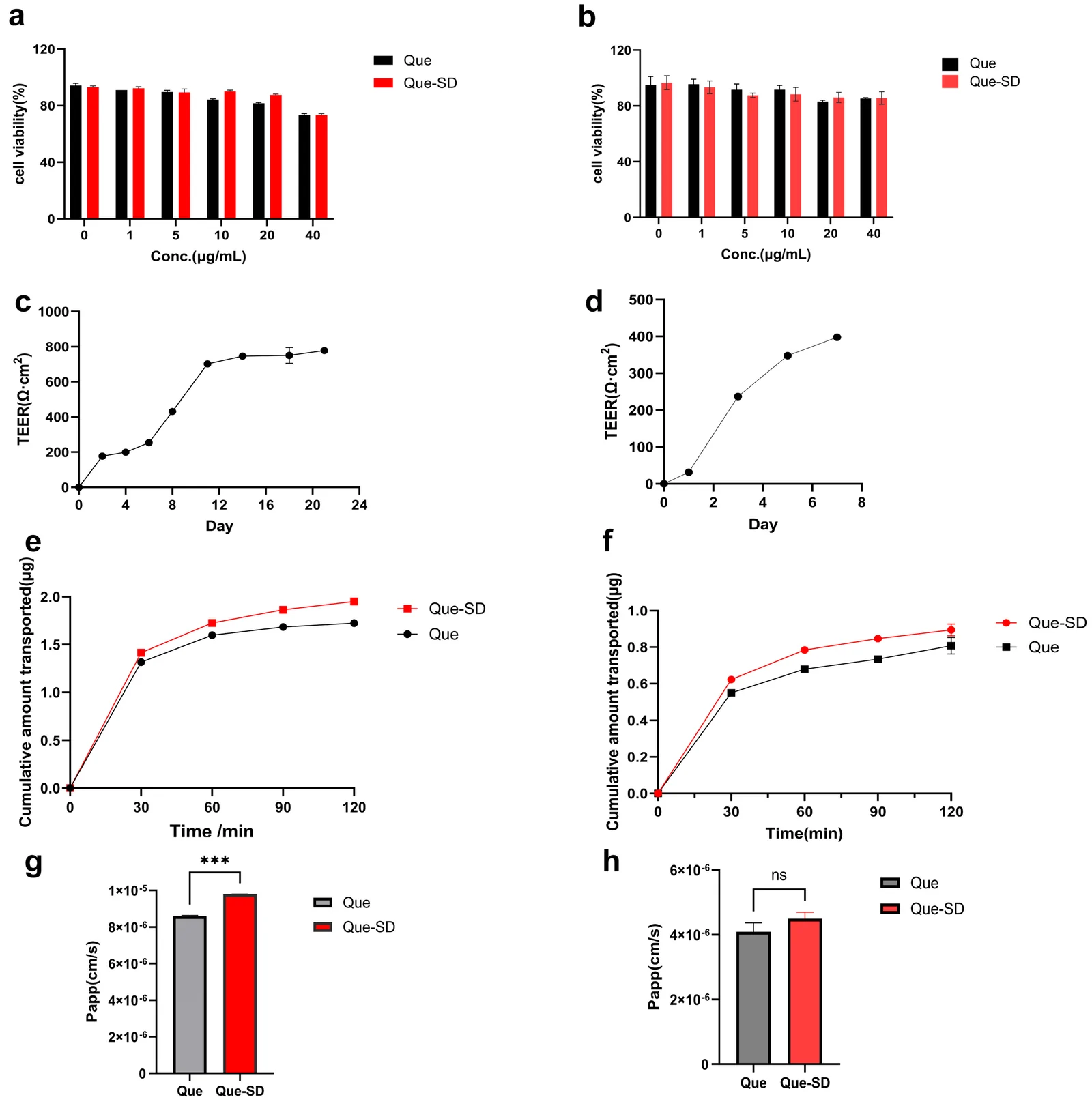
Cell viability of quercetin (QUE) and quercetin solid dispersions (QUE-SD) in Caco-2 cells (**a**) and MDCK cells (**b**). The TEER values versus time profile of Caco-2 (**c**) and MDCK (**d**) cells monolayer model. Data represent mean ± SD, (*n* = 3). Cumulative transport amount of QUE and QUE-SD in Caco-2 cell (**e**) and MDCK cell (**f**) model transports. Papp of QUE and QUE-SD transport in Caco-2 cell (**g**) and MDCK cell (**h**) monolayer models. Data represent mean ± SD, (*n* = 3). *** *p* < 0.001, ns, *p* > 0.05.

This study evaluated the cytotoxicity of QUE and QUE-SD in Caco-2 and MDCK cells using the CCK-8 method, as shown in Figure 9. Within the concentration range of 0–40 µg/mL, both formulations had a survival rate of ≥80% in MDCK cells, showing no significant cytotoxicity. However, the survival rate of both formulations in Caco-2 cells was slightly less than 80% at 40 µg/mL. Therefore, the safe concentration range was selected as 0–20 µg/mL, and 20 µg/mL was chosen for subsequent transmembrane transport experiments.

To simulate the transport process of intestinal epithelial cells for QUE and QUE-SD, Caco-2 cells and MDCK monolayer models were established. When the transmembrane resistance of Caco-2 cells is greater than 500 Ω·cm^2^, it indicates that the cell monolayer maintains complete membrane integrity, which is suitable for transmembrane transport experiments. When the transmembrane resistance of MDCK cells is greater than 200 Ω·cm^2^, the cell monolayer maintains complete membrane integrity. In this study, the TEER of the Caco-2 cell monolayer increased with the extension of culture time; on the 21st day, a dense and complete monolayer formed by Caco-2 cells was observed under a microscope, with an average TEER of (778.00 ± 2) Ω·cm^2^, meeting the standards of the Caco-2 cell monolayer model. The resistance measurement results are shown in Figure 9c. The TEER of the MDCK cell monolayer increased with the extension of culture time; on the 7th day, a dense and complete monolayer formed by MDCK cells was observed under a microscope, with an average TEER of (398 ± 3) Ω·cm^2^, meeting the standards of the MDCK cell monolayer model. The resistance measurement results are shown in Figure 9.

The cumulative transported amount of QUE and QUE-SD in cells is shown in Figure 9e,f. The experimental results show that the cumulative transported amount of QUE-SD was significantly higher than that of the QUE group in both cell types, and it was significantly higher than that of the QUE group in Caco-2 cells. This study employed the Caco-2 cell and MDCK cell transport models to conduct an in-depth investigation of the transmembrane permeability of quercetin and its solid dispersion, and the results are shown in Figure 9f. In the experiment, the apparent permeability coefficient (Papp) of 20 μg/mL quercetin in Caco-2 cells and MDCK cells was 8.60 × 10^−6^ cm/s and 4.10 × 10^−6^ cm/s, respectively, and the Papp value of the solid dispersion significantly increased to 9.80 × 10^−6^ cm/s and 4.50 × 10^−6^ cm/s, respectively, which was 1.14 and 1.1 times higher than that of the quercetin crude drug in the Caco-2 cell and MDCK models, respectively. This result indicates that the solid dispersion technology can enhance the transmembrane permeability of quercetin.

### 2.5. Pharmacokinetic Study in Rats

**Figure 10:**
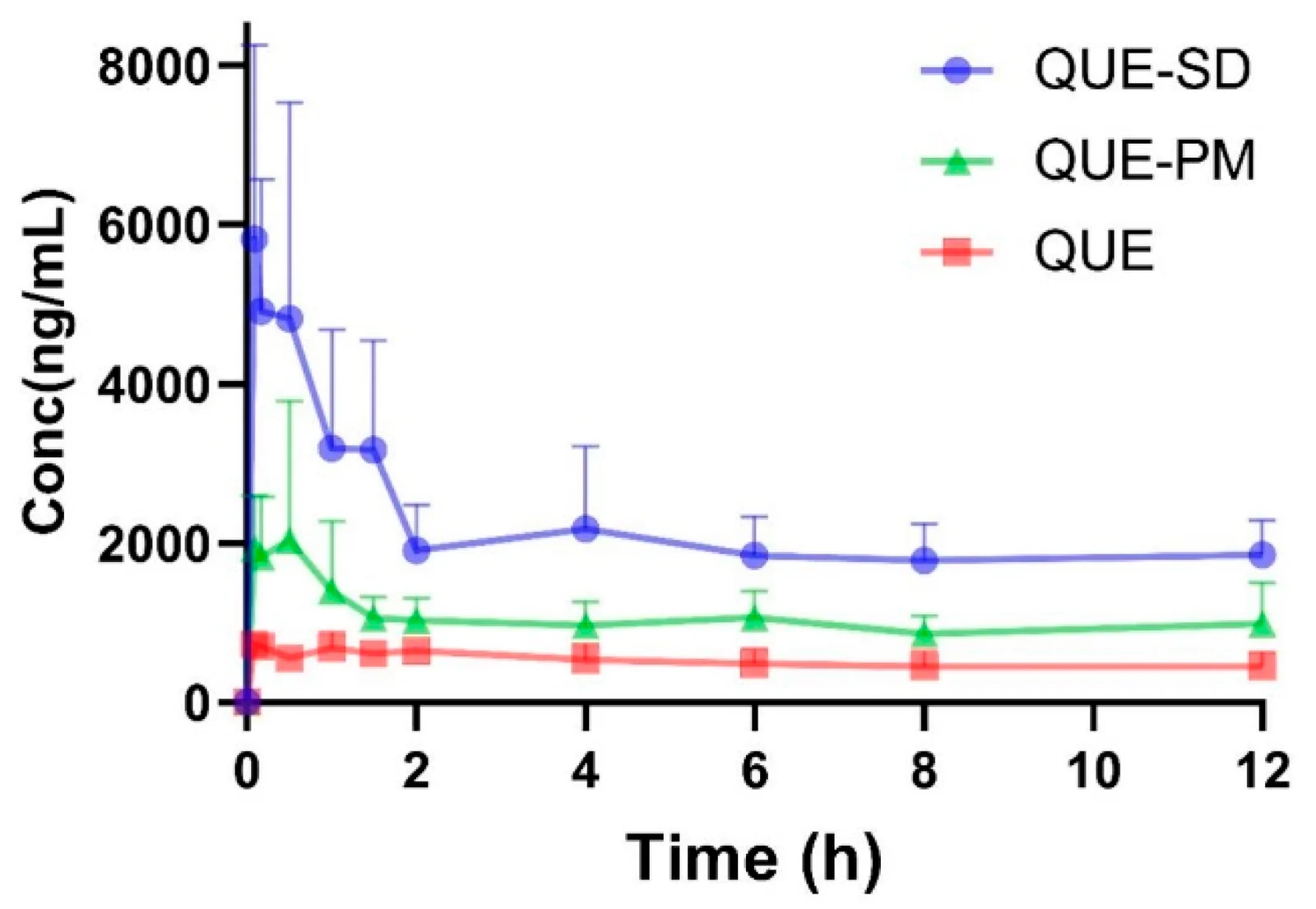
The plasma concentration of QUE following a single oral administration of QUE, QUE-PM and QUE-SD at 40 mg/kg body mass. Data present mean ± SD, (*n* = 6).

The next step was to confirm whether QUE-SD alters the pharmacokinetic profile of QUE in rats. QUE concentrations in rat plasma at different time points were determined and concentration–time curves were plotted (Figure 10). Pharmacokinetic parameters were calculated by WinNonlin^®^ (version 3.1, Pharsight, Mountain View, CA, USA) for non-compartmental analysis and statistically analyzed (Table 2). QUE-SD showed a significant increase in C_max_ and AUC_all_, while MRT and T_max_ did not change significantly. The C_max_ of QUE-SD was significantly increased compared to QUE and QUE-PM (*p* < 0.01), indicating that QUE-SD could increase the blood concentration of QUE in vivo. In addition, the CL of QUE-SD was also smaller than QUE, indicating that QUE elimination was slower in QUE-SD, while the MRT_last_ value of QUE-SD was not noticeably different from that of QUE. The relative bioavailability of QUE-SD was calculated to be 407.6%. The area under the concentration–time curve values of QUE-SD and QUE were 20,991 ± 7713 h·ng/mL versus 5150 ± 1464 h·ng/mL, respectively. The F for the physical mixture was 169.9%, indicating that the bioavailability of QUE-SD was higher than that of QUE-PM. These results were consistent with the results of in vitro dissolution studies (Figure 10) and suggested that QUE-SD can increase the solubility and dissolution rate of QUE in the gastrointestinal tract, thus improving its oral bioavailability in rats.

## 3. Discussion

The present study successfully prepared QUE-SD, and QUE was present in an amorphous state. The dissolution rate indicates the speed at which the drug is released from the carrier material. In our study, the drug dissolution rate of the QUE-SD was superior to that of the QUE-PM. The results of SEM indicate that QUE was successfully incorporated into the carrier. XRD and FT-IR affirmed that QUE-SD transformed the crystal structure of QUE [48,49], predominantly present in an amorphous or molecularly dispersed state. DSC analyses confirmed that QUE-SD altered the exothermic peaks of QUE and the carrier, indicating that QUE-SD obtained through evaporation technology can significantly enhance the dissolution rate and the solubility of QUE. Improvements in dissolution rate and solubility can lead to increased bioavailability and the absorption of QUE in the body. In this study, the drug dissolution rate of QUE-SD was better than that of QUE-PM, which suggests that by inhibiting the recrystallization of QUE through hydrogen bonding between PVP K13 and HPMC E5 to maintain the amorphous morphology (as confirmed by XRD/DSC), an increase in the specific surface area could facilitate the dissolution. Taken together, the XRD, DSC, and FTIR results suggest that the polymer matrix reduces the molecular mobility of QUE and inhibits its molecular rearrangement, nucleation, and crystal growth, thereby helping to maintain QUE predominantly in an amorphous or molecularly dispersed state. A limitation of the present study is that the physical stability of QUE-SD during storage was not evaluated. Because amorphous QUE may recrystallize over time, future studies should assess the stability of QUE-SD under defined storage conditions using XRD, DSC, and dissolution testing.

This study employed the classic intestinal epithelial cell models Caco-2 and MDCK, systematically evaluated the transmembrane permeability characteristics of QUE and its QUE-SD. Firstly, by measuring the transmembrane resistance values of the two models, the integrity and functionality of the cell monolayer were verified, and the results showed that both models meet the basic requirements for drug intestinal absorption research, providing a reliable experimental basis for subsequent permeability comparisons. In the cell transport experiments, the cumulative permeability of QUE-SD in both Caco-2 and MDCK cells was significantly higher than that of the original QUE, and this difference was more pronounced in the Caco-2 model, indicating that QUE-SD has a higher transmembrane permeability in cell models simulating human intestinal absorption. This result suggests that the solid dispersion technology can significantly improve the transmembrane transport efficiency of quercetin, thereby possibly enhancing its absorption and bioavailability in the body. Further analysis of its apparent permeability coefficient (Papp) found that the Papp values of QUE-SD in both cell models were higher than those of the QUE group. The Papp values of QUE in Caco-2 and MDCK were 8.60 × 10^−6^ cm/s and 4.10 × 10^−6^ cm/s, respectively, while those of QUE-SD were increased to 9.80 × 10^−6^ cm/s and 4.50 × 10^−6^ cm/s, respectively. These results indicate that the solid dispersion improves the permeability efficiency of QUE, possibly by further improving the drug’s solubility and dissolution rate, thus enhancing the drug’s permeability to intestinal epithelial cells and promoting drug absorption. In addition, the experimental results show that the apparent permeability coefficients (Papp) of QUE and its QUE-SD in the Caco-2 cell model were both higher than those in the MDCK cell model. This phenomenon may be attributed to the fact that Caco-2 cells are more similar to human intestinal epithelial cells in terms of physiological structure and functional characteristics. Caco-2 cells not only have more complete tight junctions and high degrees of polarization differentiation but also express various uptake transporters, such as peptide transporter 1 (PEPT1) and organic anion transporter (OATP), among others. Existing studies have shown that these transporters may be involved in the transmembrane transport processes of various flavonoid compounds. Therefore, the Caco-2 cell model shows higher sensitivity to the permeability of QUE and its solid dispersion, and it may be more suitable for evaluating the intestinal absorption behavior of flavonoid drugs.

T_max_, C_max_ and AUC are the crucial parameters for assessing the absorption process in vivo. T_max_ refers to the time required to reach the peak plasma concentration, while C_max_ denotes the highest plasma concentration after administration. Both parameters are related to the rate and extent of drug absorption following administration. AUC stands for the area under the drug concentration–time curve, reflecting the total amount of drug in the systemic circulation. The result of a pharmacokinetic study in rats showed a significant increase in both C_max_ and AUC after the oral administration of QUE-SD, with a bioavailability of 407.6%, which was higher than that of QUE (Figure 10 and Table 2). There was also an increase in AUC for QUE-PM compared to QUE. The enhancement of bioavailability may be attributed to the fact that HPMC can avoid the first-pass effect by promoting the lymphatic transport of fat-soluble components through micelle formation, and its viscosity delays gastric emptying, increases the absorption time window, and inhibits drug aggregation and maintains the supersaturation state. PVP K13 fast wetting was implemented to achieve instantaneous drug dispersion.

Thus, the therapeutic effect of QUE in the human would be enhanced because of its increased water solubility and bioavailability [50]. This would facilitate the body’s absorption of QUE. The results indicate that solid dispersion technology can enhance the release rate of QUE, thereby improving their oral bioavailability in the body.

## 4. Materials and Methods

### 4.1. Chemicals and Reagents

PVPK and HPMC series were purchased from Shanghai Macklin Biochemical Technology Co., Ltd. (Shanghai, China). Fisetin and quercetin were purchased from Shanghai Macklin Biochemical Technology Co. Formic acid and acetonitrile purchased from Merck (Darmstadt, Germany).

Caco-2 and MDCK cell lines are derived from the ATCC cell bank. Phosphate-buffered saline (PBS), HBSS buffer, DMEM medium, MEM medium, 0.25% EDTA-pancreatic enzyme, and 1% (*v*/*v*) penicillin–streptomycin are purchased from Gibco (Thermo Fisher Scientific, Grand Island, NY, USA). Fetal bovine serum is purchased from Yeasen Biotechnology (Shanghai) Co., Ltd. (Shanghai, China), while 0.4 μm Tranwell plates and 96-well cell culture plates are purchased from LABSELECT (Beijing, China).

### 4.2. Animals

Male Sprague–Dawley rats (aged up to 4 weeks) weighing approximately 300 g were housed in an animal house with free access to food and water and maintained on a 12 h light/dark cycle with controlled temperature and humidity. The rats were fasted for at least 12 h before drug administration but were allowed free access to water. Animal experiments were approved by the Institutional Animal Care and Use Committee of the Ministry of Science and Technology by the Regulations on the Management of Laboratory Animals.

### 4.3. Preparation of Solid Dispersions and Physical Mixtures

The solid dispersion of QUE was prepared by the solvent evaporation method. The specified amount of QUE was accurately weighed, added to 20 mL of anhydrous ethanol, and ultrasonicated for 20 min until completely dissolved. The corresponding amounts of HPMC E5 and PVP K13 were accurately weighed and slowly added to 30 mL of anhydrous ethanol until dissolved. The drug and carrier solutions were mixed thoroughly and stirred for 4 h to ensure uniform dispersion of QUE within the carrier system. The solvent was then removed by rotary evaporation at 55 °C, and the resulting product was dried at 50 °C in a forced-air drying oven. After drying, the solid dispersion was ground and passed through an 80-mesh sieve.

The drug and carrier were weighed in the ratio of QUE, HPMC E5 and PVP-K13 1:3:1 and ground in a mortar and pestle for 20 min. Physical mixtures of physical mixture of quercetin physical mixture (QUE-PM) obtained were passed through an 80-mesh sieve to prepare a physical mixture.

### 4.4. Formulation Screening and Preparation Process Optimization

QUE-SD was prepared by the solvent evaporation method. The formulation screening consists of two stages: single-carrier screening and combined-carrier screening. In the single-carrier screening stage, the drug and each carrier were accurately weighed at the mass ratio of QUE to carrier (PVE/PEG) of 1:4 and QUE to HPMC of 1:2, respectively.

In the combined-carrier screening stage, after HPMC E5 and PVP K13-18 were selected as the combined carriers, formulations with the mass ratios of QUE: E5: K13-18 at 1:1:1, 1:1:2, 1:2:1, 1:1:3, 1:3:1 and 1:2:2 were further investigated, and the mass ratio of 1:3:1 was finally determined as the formulation proportion for subsequent studies.

QUE orthogonal experimental design: orthogonal table L9 was shown to design orthogonal experiments to select the best preparation process for quercetin solid dispersion (QUE-SD). As shown in Table 3, the design was based on two factors for three levels of QUE, HPMC E5, and PVP-K13: Stirring time (A) and rotary evaporation temperature (B).

### 4.5. Characterization of Solid Dispersions

#### 4.5.1. Scanning Electron Microscopy (SEM)

A small amount of the prepared sample was dipped onto a toothpick and scattered on the sample base with conductive tape, sprayed with gold for 5 min, then removed and observed on a Sigma 300 scanning electron microscope (Carl Zeiss Microscopy GmbH, Jena, Germany) at a constant voltage of 15.0 kV.

#### 4.5.2. X-Ray Diffraction (XRD)

Drug crystallinity was determined using an XRD-6000 diffractometer (Shimadzu Corporation, Kyoto, Japan) equipped with Cu-Kα at 40 kV and 35 mA. The scanning speed was 1°/min, the scanning range was from 5° to 35° at room temperature, and the wavelength was set at 1.54184 α.

#### 4.5.3. Fourier Transform Infrared Spectroscopy (FT-IR)

The sample was mixed with KBr and ground uniformly in an agate mortar. The ground mixture was poured into a mold press. After pressing the QUE-SD sample, the 4000–400 cm^−1^ spectral region was tested using an infrared spectrometer (IR Prestige-21, Shimadzu Corporation, Kyoto, Japan) with a resolution of 2 cm^−1^.

#### 4.5.4. Differential Calorimetric Scanning (DSC)

DSC and TGA measurements of QUE, carriers, physical mixtures, and solid dispersions were carried out using a TGA-DSC simultaneous thermogravimetric analyzer. The 5.0–10.0 mg samples were placed in an aluminum pot with a nitrogen flow rate of 50 mL/min and a heating rate of 10 °C/min from 20 °C to 550 °C. The samples were then heated from 20 °C to 550 °C at a flow rate of 10 °C/min.

### 4.6. HPLC Analysis of Quercetin

The analysis was performed on an Agilent 1260 High-Performance Liquid Chromatograph (Agilent Technologies, Santa Clara, CA, USA) using a Waters Ultimate C18 column (4.6 mm× 150 mm, 5 μm) with a mobile phase of methanol: 0.1% phosphoric acid solution (containing 0.2% triethylamine) = 50:50 (filtration through 0.45 μm microporous membrane).

### 4.7. Solubility and Cumulative Dissolution Determination

The solubility of QUE and different QUE-SD formulations in distilled water was determined using the following method. Samples equivalent to 10 mg of QUE were accurately weighed and placed into centrifuge tubes each containing 20 mL of distilled water. The mixtures were shaken at 37 °C for 24 h, filtered through a 0.45 μm microporous membrane filter, and the QUE concentration in the filtrate was determined by the aforementioned HPLC method.

In vitro release of QUE was evaluated using the paddle method with the RC-806D (Tianjin Tianda Tianfa Technology Co., Ltd., Tianjin, China) dissolution apparatus according to the Chinese Pharmacopoeia. Before the experiment, 500 mL of distilled water was poured into a dissolution cup as the dissolution medium at 37 ± 0.5 °C with a stirring paddle speed of 50 rpm. At the beginning of the experiment, QUE, QUE-PM and QUE-SD (equivalent to 10 mg of QUE) were added to the dissolution medium and aspirated 5 mL of the sample at 10, 20, 30, 50, 70, 90, 120, 150, 180, 210 and 240 min. The samples were filtered through a 0.45 μm membrane filter; the filtrate was analyzed by HPLC.

### 4.8. Cell Test

#### 4.8.1. Cell Culture

Culture Caco-2 cells in MEM medium containing 10% fetal bovine serum (*v*/*v*) and 1% penicillin–streptomycin, and change the medium every 2–3 days. Culture MDCK cells in DMEM medium containing 5% fetal bovine serum and 1% penicillin–streptomycin, and change the medium every 1–2 days. Cultivate the cells in an incubator at 37 °C, 5% CO_2_, and 95% relative humidity. Prepare the samples with DMSO and dilute to different concentrations of QUE and QUE-SD with Hank’s balanced salt solution (HBSS, PH = 7.4).

#### 4.8.2. Cytotoxicity Test

Caco-2 and MDCK cells were separately seeded into 96-well plates at a density of 5 × 10^3^ cells/well (100 μL/well) and cultured in an incubator at 37 °C, 5% CO_2_, and 95% relative humidity for 24 h. After discarding the culture medium, a fresh medium containing different concentrations of QUE or QUE-SD (100 μL/well) was added, and the cells were further cultured for 24 h. Subsequently, the medium was discarded, and the cells were gently washed with PBS 2–3 times. Ten percent (*v*/*v*) CCK-8-containing medium (100 μL/well) was added to each well. Caco-2 cells were incubated for 2 h, and MDCK cells were incubated for 0.5 h. The absorbance (A) of each well was measured at 450 nm using an enzyme-linked immunosorbent assay (ELISA) reader, and cell viability was calculated using the following formula.

Cell vilability (% of control) = (SampleP − SampleB)/(SampleN − SampleB) × 100%(1)

The cell viability in the formula (percentage of control) is the cell survival rate; SampleP is the OD value of the positive control group; SampleN is the OD value of the negative control group; SampleB is the OD value of the blank control group.

#### 4.8.3. Cell Monolayer Transport Test

To evaluate cell permeability, establish a cell monolayer transport model, and seed Caco-2 (1 × 10^5^ cells/well) or MDCK (5 × 10^4^ cells/well) in a 12-well Transwell plate. Add 0.5 mL cell suspension to the apical (Apical, AP) side of the Transwell plate, and 1.5 mL complete culture medium to the basolateral (Basolateral, BL) side. Cultivate the plate at 37 °C, 5% CO_2_, and saturated humidity. Cultivate Caco-2 for 21 days (medium changed every other day), and MDCK for 7 days (medium changed daily). Measure the trans-epithelial electrical resistance (Trans-Epithelial Electrical Resistance, TEER, Beijing Jinguang Hongtai RE1600, Beijing, China) using an epithelial cell resistance meter to evaluate the integrity of the cell monolayer. When the Caco-2 cell resistance is >500 Ω·cm^2^, and the MDCK cell resistance is >200 Ω·cm^2^ [51], proceed with the subsequent cell monolayer transport experiments.

Based on the results of the preliminary cytotoxicity tests, determine the safe dosage concentration range. Add the drug to the Transwell apical chamber (AP) and add 1.5 mL preheated HBSS (pH 7.4) to the basolateral side (BL). Incubate at 37 °C, 5% CO_2_, and at 30, 60, 90, and 120 min, respectively, withdraw 200 µL samples from the BL chamber, and immediately replenish with the same volume of blank HBSS. Take 100 µL of the sample, sequentially add 20 µL of 0.5% (*w*/*v*) sodium ascorbate and 50 µL of methanol, vortex for 5 min, then centrifuge at 13,000 rpm, 4 °C for 5 min, and take 20 µL of the supernatant for analysis. The drug concentration is detected by HPLC, and the apparent permeability coefficient (Papp) is calculated according to the following formula: Papp = (dQ/dt)/(A·C0).

In the formula, dQ/dt is the osmotic rate per unit time; A is the surface area of the polycarbonate membrane (1.12 cm^2^); C0 is the initial concentration of the drug (μg/mL).

### 4.9. Animals Study

#### 4.9.1. Pharmacokinetic Study Design

Eighteen healthy Sprague–Dawley rats were randomly divided into three groups of six rats each: QUE group, QUE-PM group and QUE-SD group. The rats were anesthetized by intramuscular injection using Zoletil (Virbac, Carros, France) and underwent femoral arteriovenous intubation. Each group of rats received a dose of 40 mg/kg of QUE, QUE-PM, and QUE-SD (containing equivalent clinical dose). Blood was collected from the femoral artery at approximately 400 μL and the same amount of saline was given in the femoral vein at 0.0833, 0.167, 0.5, 1, 1.5, 2, 4, 6, 8, and 12 h after oral gavage administration, respectively. The obtained blood samples were immediately centrifuged, and the plasma supernatant was collected and stored in a −80 °C until UPLC-MS/MS analysis.

#### 4.9.2. Preparation of Plasma Sample

For a 50 μL of plasma sample added into a 1.5 mL centrifuge tube, add 10 μL of 2% formic acid, 10 μL of 10 mg/mL sodium ascorbate, followed vortex mixing for 1 min. Then add 5 μL of 250 μg/mL laccase and 200 μL of acetonitrile, followed vortex mixing for 10 min. When centrifugating the supernatant for 10 min at 4 °C, centrifugation speed was set as 13,000 rpm and the supernatant was analyzed by UPLC-MS/MS [52].

#### 4.9.3. UPLC-MS/MS Analysis

All plasma samples were analyzed by UPLC-MS/MS (6495C, Agilent Technologies, Inc., Santa Clara, CA, USA). using a Welch Ultimate XB C18 (150 mm × 4.6 mm, 5 μm) column. The mobile phase was acetonitrile-1% formic acid water with gradient elution at a flow rate of 0.8 mL/min. MS/MS analysis was performed on an Agilent 6495C triple quadrupole mass spectrometer with an electrospray ionization (ESI) technique. The analysis was carried out in negative ion ESI mode with drying gas temperature at 250 °C and a sheath gas flow rate of 12.0 L/min, as well as with nitrogen temperature at 350 °C and a nitrogen flow rate of 11.0 L/min. The monitored ion pairs of QUE and the internal standard were *m*/*z* 301.0 → 251.0 and *m*/*z* 284.9 → 121.0, respectively.

### 4.10. Data Analysis

Relative bioavailability was calculated as follows. AUCT is the area under the curve for QUE-SD dosing time and AUCR is the area under the curve for QUE dosing time, and D denotes the administered dose.

F = (AUCT × DR)/(AUCR × DT) × 100%(2)

Pharmacokinetic parameters were calculated using WinNonlin software (version 3.1, Pharsight, Mountain View, CA, USA) with a non-compartmental model.

## 5. Conclusions

The optimal formulation was screened by a two-factor, three-level orthogonal test, i.e., the ratio of QUE, HPMC and PVPK was 1:3:1, the temperature was 55 °C and the stirring time was 4 h. Characterization by X-ray diffraction, scanning electron microscopy, and FTIR spectroscopy showed that amorphous solid dispersions had been formed. QUE-SD significantly improved the dissolution rate and oral bioavailability of QUE. This study will provide a new preparation method and an idea for the preparation of oral QUE solid dispersion formulations.

## Figures and Tables

**Table 1:** Orthogonal design and result table.

Sample	A	B	Cumulative Dissolution Rate (%)
1	1	1	88.8
2	1	2	84.9
3	1	3	101.0
4	2	1	92.4
5	2	2	80.4
6	2	3	100.1
7	3	1	85.5
8	3	2	80.2
9	3	3	83.6
K_1_	91.6	88.9	
K_2_	90.9	81.8	
K_3_	83.1	94.9	
R	8.5	6.0	

Note: The maximum range method was used to process the data and select the best combination. K_1_, K_2_, and K_3_ denote the sum of the indices at the level of each factor, and R denotes the maximum difference between these three parameters.

**Table 2:** Pharmacokinetic parameters after a single oral administration of QUE, QUE-PM and QUE-SD (Mean ± SD, *n* = 6).

Pharmacokinetic Parameters	QUE	QUE-PM	QUE-SD
T_max_ (h)	0.1 ± 0.4	0.3 ± 0.2	0.5 ± 0.7
C_max_ (ng/mL)	771 ± 121	2081 ± 1349	7127 ± 1942 **
t_1/2_ (h)	38 ± 29	17 ± 10	9 ± 3
AUC_all_ (h·ng/mL)	5150 ± 1464	8751 ± 2908 *	20,991 ± 7713 *
VZ (mL/kg)	70,096 ± 24,073	33,622 ± 16,302 **	12,159 ± 2298 *
CL (mL/h·kg)	2219 ± 1378	1793 ± 1019	1002 ± 419
MRTlast (h)	4.6 ± 0.3	4.4 ± 0.4	3.9 ± 1.2
F (%)	-	169.9	407.6

Note: T_max_ and C_max_ were indicate time to peak and peak concentration, respectively; AUC_all_ is the area under the drug–time curve; VZ and CL indicate the apparent volume of distribution and clearance, respectively; MRTlast, mean residence time from the time of dosing to the time of the last measurable plasma concentration, calculated as AUMC_last_/AUC_last_. and F is the relative bioavailability. *p*-values are the paired *t*-test analyses of pharmacokinetic parameters for the QUE-SD, QUE-PM, and QUE control groups. * indicates *p* < 0.05 when compared with QUE, ** indicates *p* < 0.01 when compared with QUE.

**Table 3:** Factors and levels of orthogonal design.

Level	A (h)	B (°C)
1	4	35
2	12	45
3	24	55

## Data Availability

The raw data supporting the conclusions of this article will be made available by the authors on request.
